# A Hybrid Convolutional Neural Network–Long Short-Term Memory (CNN–LSTM)–Attention Model Architecture for Precise Medical Image Analysis and Disease Diagnosis

**DOI:** 10.3390/diagnostics15212673

**Published:** 2025-10-23

**Authors:** Md. Tanvir Hayat, Yazan M. Allawi, Wasan Alamro, Salman Md Sultan, Ahmad Abadleh, Hunseok Kang, Aymen I. Zreikat

**Affiliations:** 1 Innovative Skills Ltd., Dhaka 1207, Bangladesh; tanvir@innovativeskillsbd.com (M.T.H.); salman@innovativeskillsbd.com (S.M.S.); 2 Department of Electrical Engineering, College of Engineering, Princess Nourah bint Abdulrahman University, P.O. Box 84428, Riyadh 11671, Saudi Arabia; 3 Department of Communications and Computer Engineering, Faculty of Engineering, Al-Ahliyya Amman University, Amman 19111, Jordan; w.alamro@ammanu.edu.jo; 4 CS Department, Mutah University, Karak 61710, Jordan; ahmad_a@mutah.edu.jo; 5 College of Engineering and Technology, American University of the Middle East, Egaila 54200, Kuwait; hunseok.kang@aum.edu.kw (H.K.); aymen.zreikat@aum.edu.kw (A.I.Z.)

**Keywords:** attention mechanism, convolutional neural networks, long short-term memory, medical image analysis, sequential features

## Abstract

**Background:** Deep learning (DL)-based medical image classification is becoming increasingly reliable, enabling physicians to make faster and more accurate decisions in diagnosis and treatment. A plethora of algorithms have been developed to classify and analyze various types of medical images. Among them, Convolutional Neural Networks (CNNs) have proven highly effective, particularly in medical image analysis and disease detection. **Methods:** To further enhance these capabilities, this research introduces MediVision, a hybrid DL-based model that integrates a vision backbone based on CNNs for feature extraction, capturing detailed patterns and structures essential for precise classification. These features are then processed through Long Short-Term Memory (LSTM), which identifies sequential dependencies to better recognize disease progression. An attention mechanism is then incorporated that selectively focuses on salient features detected by the LSTM, improving the model’s ability to highlight critical abnormalities. Additionally, MediVision utilizes a skip connection, merging attention outputs with LSTM outputs along with Grad-CAM heatmap to visualize the most important regions of the analyzed medical image and further enhance feature representation and classification accuracy. **Results:** Tested on ten diverse medical image datasets (including, Alzheimer’s disease, breast ultrasound, blood cell, chest X-ray, chest CT scans, diabetic retinopathy, kidney diseases, bone fracture multi-region, retinal OCT, and brain tumor), MediVision consistently achieved classification accuracies above 95%, with a peak of 98%. **Conclusions:** The proposed MediVision model offers a robust and effective framework for medical image classification, improving interpretability, reliability, and automated disease diagnosis. To support research reproducibility, the codes and datasets used in this study have been publicly made available through an open-access repository.

## 1. Introduction

### 1.1. Overview

The integration of Machine Learning (ML) into medical practice has revolutionized the healthcare field, enabling computers to analyze vast amounts of patients data and derive insights into solving complex clinical problems without explicit programming. As a subset of ML specialized in utilizing artificial neural networks with multiple layers to analyze data, Deep Learning (DL) is playing an increasingly important role in enhancing treatment strategies for chronic conditions, supporting precision medicine, and expediting clinical trials [1,2] and promising significant advances in areas such as diagnostic processes [3,4,5], prognosis prediction [6,7,8], and drug discovery [4,5].

Among these applications, medical image classification presents unique challenges due to various factors. The variability in medical images, due to differences in imaging techniques, patient demographics, and the presence of artifacts, complicates the development of reliable and robust models [9,10]. To elaborate, medical images often contain subtle yet critical details that are essential for accurate diagnosis, requiring highly sensitive and precise models. In parallel, tabular medical data, including patient records, lab results, and other clinical information, presents additional challenges [11,12]. Such data are typically high-dimensional, may contain missing values, and are subject to strict privacy regulations, all of which complicate the classification process [13]. Various ML, DL, and hybrid algorithms, such as Convolutional Neural Networks (CNNs), Long Short-Term Memory (LSTM) networks, Generative Adversarial Networks (GANs), and Support Vector Machine (SVM) [14,15,16,17,18], have advanced automated medical image analysis by improving classification accuracy. However, existing models still face challenges in feature extraction, sequential pattern recognition, generalization across datasets, and computational efficiency [19,20,21].

### 1.2. Advances in CNN-Based Medical Image Classification

Medical image analysis has significantly benefited from the advancements in CNNs, as they are highly effective for feature extraction, which is essential for accurate disease detection. The work by P. M. de Sousa et al. in [22] explored Wavelet CNNs, demonstrating improved CT image integrity without requiring data augmentation, resizing, or segmentation techniques. Other CNN-based architectures like C-Net [23] and Convolutional Deep Belief Networks (CDBN) [24,25] have also shown promise in medical image classification. The C-Net model consists of a concatenation of three main components: Outer, Middle, and Inner networks. The Outer and Middle networks function as feature extractors, while the Inner network is responsible for classifying the images as malignant or benign; whereas the CDBN model utilizes SVM as a feature classifier dedicated to classifying CT images to enhance feature transfer and reuse while suppressing less useful ones.

### 1.3. Emerging Hybrid Architectures for Enhanced Feature Representation

Recognizing the inherent limitations of CNNs in capturing global context and sequential dependencies, several hybrid models have been explored for medical image analysis. Among these are the models that proposed combining CNNs with Transformers, such as MambaConvT [26], which utilizes Transformers for enhanced global context. Other hybrid approaches have incorporated techniques like autoencoders and Particle Swarm Optimization (PSO) [27] or ensemble learning methods (e.g., Random Forest and XGBoost) [28] to optimize feature selection and improve classification performance. Further advancements include the integration of EfficientNet with SVM [29] and the development of Hybrid Quantum CNNs (HQCNNs) with adaptive optimizers [30]. Transfer learning with pretrained CNNs has been explored in [31], enabling deep feature extraction that is more abstract and representative of underlying data patterns compared to traditional methods.

Recent advances continue to emphasize the synergy between CNNs and Vision Transformers (ViTs) for improved medical image analysis. Borji et al. [32] introduced a hybrid CNN-ViT-MLP model tailored for osteosarcoma histopathology image classification, achieving high performance on a four-class Cancer Imaging Archive (TCIA) dataset. Their model effectively captured both local and global features without relying on segmentation, highlighting the strength of combining CNNs for fine-grained pattern recognition and ViTs for long-range dependencies. Similarly, the work in [33] proposed the EFFResNet-ViT framework, which fuses EfficientNet-B0 and ResNet-50 backbones with ViT modules. The model demonstrated strong performance on brain tumor MRI and retinal image datasets, while also emphasizing model interpretability via Grad-CAM visualizations and t-SNE analysis. These works underline a growing trend in hybrid architectures that balance accuracy and explainability, which are considered vital requirements for clinical integration. Nonetheless, despite these promising results, the current models have primarily been evaluated on limited datasets and may lack the generalizability and diversity required for broader clinical applications, highlighting the need for further investigation in more varied and extensive data contexts.

### 1.4. Sequential and Attention-Based Models for Context-Aware Analysis

Recently, advancements in medical image classification resulted in the development of hybrid models that combine CNNs with RNNs, such as LSTM, which are capable of capturing both spatial and temporal features, such that CNNs excel at extracting local spatial features, while LSTM are designed to handle sequential data, making them suitable for learning temporal patterns. For instance, Salehin et al. [34] introduced MedvLSTM, a hybrid model that integrates CNNs and LSTM to enhance feature representation and classification accuracy in medical image analysis. Complementing these efforts, attention-based models have emerged as powerful tools to improve both accuracy and efficiency in medical imaging tasks. These models utilize attention mechanisms to prioritize relevant features within images, leading to superior classification outcomes across diverse datasets as in [35], where the integration of spatial attention in dual-input CNNs has demonstrated the potential to effectively fuse features from original and modified images. Further innovations in attention mechanisms have expanded their applicability to multi-scale and segmentation tasks. Cheng et al. [36] proposed the Attention-based Multi-scale Nested Network (AMNNet), which incorporates Convolutional Block Attention Modules (CBAM) to capture contextual information at varying scales, highlighting the versatility of attention mechanisms in both segmentation and classification. Similarly, Liu et al. [37] introduced Eff-CTNet, a model that combines CNNs and Transformer architectures with a Group Cascade Attention module, capable of reducing computational costs while maintaining high performance on small-scale datasets, and thus balancing between efficiency and accuracy in medical image analysis.

### 1.5. Motivation and Research Gaps

The comprehensive literature review presented above underscores the growing importance of hybrid approaches in advancing medical image classification. Techniques such as CNN-LSTM integration, attention mechanisms, and multi-scale feature fusion have demonstrated significant potential in addressing the challenges posed by complex medical datasets. However, while these methods have achieved notable progress, limitations remain, particularly in terms of interpretability, computational efficiency, and robustness across diverse datasets. Existing models often struggle to balance these factors effectively, emphasizing the need for a more unified and adaptive solution. In response to these challenges, we propose the MediVision model, a novel framework that seamlessly integrates the strengths of CNNs, LSTM, attention mechanisms, and interpretability tools like Grad-CAM heatmaps. By combining these key components, MediVision not only addresses the limitations of current models but also sets a new benchmark for performance and transparency in medical image analysis. In the following section, we detail the architecture and methodology of MediVision, illustrating how its innovative design achieves state-of-the-art results while ensuring interpretability and scalability.

**Figure 1:**
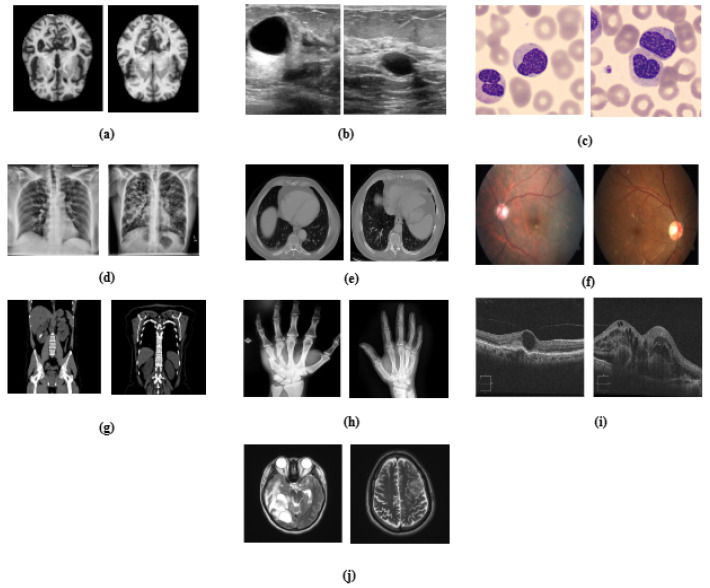
Visual representation of medical datasets used in the study, illustrating sample images for (**a**) Alzheimer’s disease, (**b**) breast ultrasound, (**c**) blood cell, (**d**) chest X-ray, (**e**) chest CT scans, (**f**) diabetic retinopathy, (**g**) kidney diseases, (**h**) bone fracture multi-region, (**i**) retinal OCT, and (**j**) brain tumor.

In this paper, we present MediVision, a hybrid CNN-LSTM-attention model specifically designed for medical image classification. Unlike prior models that simply combine CNNs and LSTMs, MediVision integrates three key components: (1) CNNs for extracting spatial features from medical images, (2) an LSTM module that captures sequential dependencies across spatial feature maps, and (3) a tailored attention mechanism applied post-LSTM to selectively emphasize clinically relevant regions. Additionally, a skip connection preserves original CNN features alongside the LSTM–attention pathway, and Grad-CAM visualization enhances interpretability, allowing clinicians to better understand decision-relevant regions. Our proposed model was extensively evaluated over ten diverse real-world medical image datasets, as illustrated in Figure 1, with performance comparison to existing models. The results demonstrate the superior performance of MediVision in achieving classification accuracies for medical image analysis and disease detection.

The main contributions of this paper are summarized as follows:

- Present a comprehensive CNN-LSTM-Attention hybrid model for medical image classification;
- Demonstrate the effectiveness and generalizability of MediVision across ten diverse medical imaging datasets;
- Investigate the ability of MediVision to seamlessly integrate and exploit spatial, temporal, and attention-based features;
- Showcase the scalability and adaptability of MediVision for a broad range of medical image analysis tasks;
- Conduct extensive comparative and statistical analyses across diverse medical imaging datasets to validate the consistency, reliability, and generalizability of MediVision.

## 2. Materials and Methods

The proposed methodology comprises multiple cascaded stages, such that it begins with dataset partitioning, followed by data augmentation, preprocessing, and then model training. Each medical dataset is first split into training (70%), validation (15%), and testing (15%) subsets to prevent data leakage and ensure unbiased evaluation. Subsequently, data augmentation is applied to the training and validation sets to increase the sample diversity and address class imbalance, while the test set remains untouched. After augmentation, the data undergo preprocessing steps including normalization and label encoding. The proposed hybrid model is then trained on the augmented training set, with performance monitored using the validation set. Final evaluation is conducted on the independent test set using standard metrics (i.e., accuracy, precision, recall, and F1-score) to assess both predictive performance and model resilience.

### 2.1. Dataset Description and Customization

The effectiveness of DL models in medical image analysis heavily relies on the quality and diversity of the datasets used for training, validation and testing. Ten diverse medical imaging datasets were utilized in this study, as summarized in Table 1, encompassing a wide range of medical conditions, including neuro-degenerative disorders, oncological abnormalities, and pulmonary diseases, which provides a comprehensive foundation for robust model use in medical image classification. These datasets were chosen as representatives of various imaging modalities and diagnostic challenges, enabling a thorough evaluation of the proposed framework. While our experiments focus on these conditions, MediVision is a generalizable framework that can be applied to a broader set of diseases with appropriate training data.

### 2.2. Data Augmentation and Normalization

**Figure 2:**
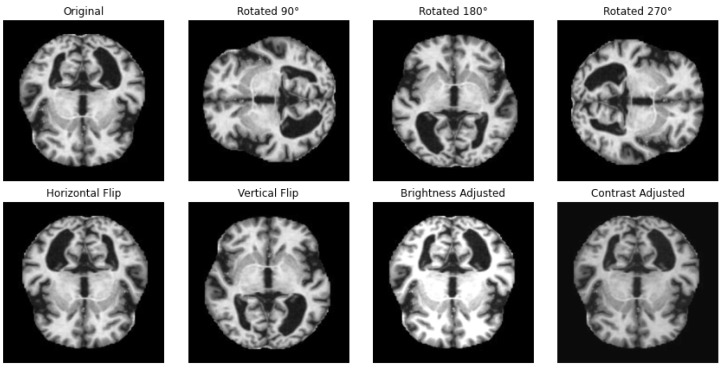
Illustration of applied data augmentation techniques.

One way to artificially expand the amount and complexity of current data is through data augmentation [53]. To give developers access to more representative training data, data augmentation techniques have been used to increase the size of training sets [54,55]. In this study, we have applied different techniques for data augmentation to enhance the diversity of the datasets and to achieve a balanced distribution of classes, as in the example illustrated in Figure 2. The augmentation methods that we have employed include rotation, horizontal and vertical flipping, and other transformations to simulate different perspectives and conditions with the parameters summarized in Table 2.

In image processing and ML-based approaches, normalizing pixel values is a standard preprocessing step. This process scales the pixel values of an image to a standardized range, typically between 0 and 1 [56]. This normalization is essential, because images are often represented in an 8-bit per channel format, where each pixel can range from 0 (black) to 255 (white) [57]. The range of pixel values is given in the closed interval [0,255]. The normalization equation is as follows:

(1) $$X_{\mathrm{normalized}}=\frac{X-X_{\min}}{X_{\max}-X_{\min}},$$

where *X* represents the pixel value, and $X_{\min}$ and $X_{\max}$ are the minimum and maximum pixel values, respectively.

### 2.3. The Proposed MediVision Model

**Figure 3:**
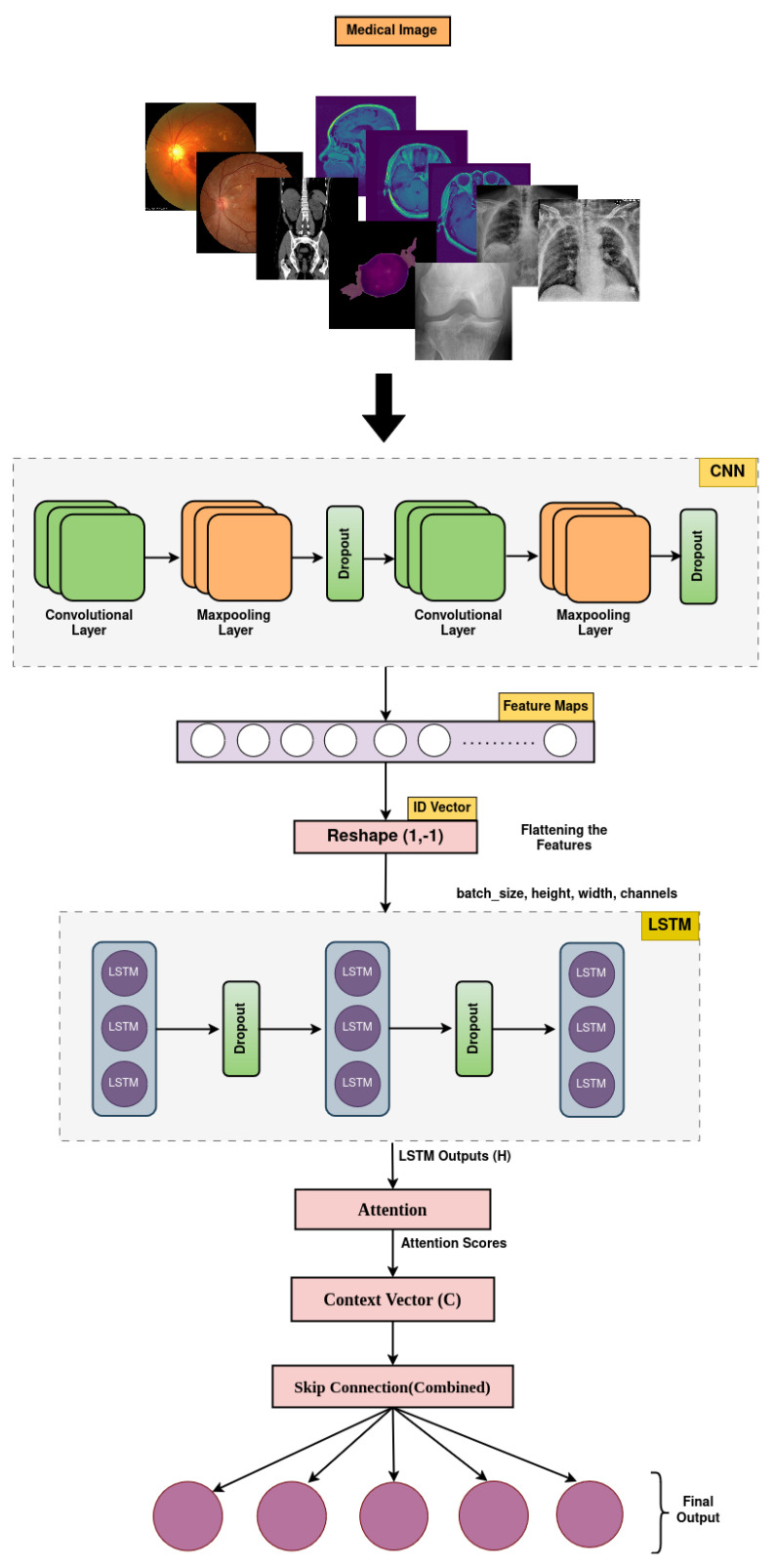
Proposed MediVision architecture.

The MediVision model is a hybrid DL-based architecture, designed to combine the strengths of CNNs, LSTM, and attention mechanism, as shown in Figure 3. This combination allows the model to effectively process and analyze visual data, while capturing sequential dependencies and focusing on the most relevant parts of the input sequence. Additionally, a feature selection component is incorporated to refine the learned representations by prioritizing the most informative features and reducing redundancy, thereby enhancing the classification performance and model interpretability. The proposed MediVision model, with details of each block illustrated in Figure 3, is described as follows.

#### 2.3.1. CNNs Unit

The convolution operation involves sliding a filter (also called a kernel) over an input to produce a feature map. This operation helps detect and preserve spatial hierarchies in images, allowing the learning of features like edges, textures, and more complex patterns [58]. Equation (2) indicates feature maps of a CNN layer.

(2) $$f_{ij}^{k}=\sigma \left(\sum_{m=0}^{M-1}\sum_{n=0}^{N-1}w_{mn}^{k}x_{\left(i+m)(j+n\right)}+b^{k}\right),$$

where $f_{ij}^{k}$ is the output feature map at position (i,j) for the *k*-th filter, $w_{mn}^{k}$ is the weight at position (m,n) in the *k*-th filter, $x_{\left(i+m)(j+n\right)}$ is the input at position (i+m,j+n), $b^{k}$ is the bias for the *k*-th filter, and σ is the ReLU activation function.

#### 2.3.2. Flattening and Reshaping Unit

Flattening and reshaping are essential preprocessing steps in this model architecture, bridging the gap between convolutional feature extraction and sequential analysis using LSTM and attention mechanisms. Flattening converts multidimensional feature maps into a format suitable for further processing, while reshaping tailors the data for sequential LSTM input, optimizing the model’s ability to capture sequential dependencies within the task of image classification. A tensor *x* after convolutional and pooling layers has the following shape:

$$x\in R^{\left(batch\_size,height,width,channels\right)}.$$

The flattening operation reshapes *x* into a 1D vector *V*:

(3) V=flatten(x),

where

$$V\in R^{\left(batch\_size,height\cdot width\cdot channels\right)}.$$

The reshaping operation prepares the data for input into the LSTM layer, which expects sequences of vectors. After flattening, *V* is reshaped into a 3D tensor *Y*:

(4) Y=reshape(V,batch_size,1,height,width,channels),

where

$$Y\in R^{\left(batch\_size,1,height,width,channels\right)}.$$

Here, 1 indicates that each input sequence to the LSTM consists of a single time step, ensuring compatibility with sequential processing. When replaced by −1, this will indicate that the reshaping operation automatically determines the remaining dimension size based on the total elements of the flattened vector.

#### 2.3.3. LSTM Unit

The LSTM layer in this model processes reshaped input data *Y* consisting of sequences of feature vectors. It computes hidden states that capture sequential dependencies within each sequence, thus giving it the ability to remember information over long periods.

(5) H=LSTM(Y),

where

$$H\in R^{\left(batch\_size,1,\mathrm{Units}\right)}.$$

Here, *H* contains the hidden states for each sequence element (time step). The LSTM computes these hidden states using a set of learned weights and biases, which are updated during training to capture sequential dependencies in the data. The LSTM processes each time step *t* using the following gate mechanisms:

(6) $$f_{t}=\sigma \left(W_{f}\left[h_{t-1},x_{t}\right]+b_{f}\right).$$

The forget gate $f_{t}$ decides how much of the previous cell state $C_{t-1}$ to retain for the current time step *t* based on the previous hidden state $h_{t-1}$ and the current input $x_{t}$, which takes the input as described in (7) to (9) below:

(7) $$I_{t}=\sigma \left(W_{i}\left[h_{t-1},x_{t}\right]\right),$$

(8) $${\tilde{c}}_{t}=\tanh\left(W_{i}\left[h_{t-1},x_{t}\right]+b_{i}\right),$$

(9) $$c_{t}=f_{t}\cdot C_{t-1}+I_{t}\cdot {\tilde{c}}_{t},$$

where the input gate $I_{t}$ determines how much of the candidate values ${\tilde{c}}_{t}$ should be added to the cell state $c_{t}$. The candidate cell state ${\tilde{c}}_{t}$ represents the new candidate values that could potentially be added to the cell state $c_{t}$. The updated cell state $c_{t}$ combines the decision of the forget gate $f_{t}$ on what to forget from $C_{t-1}$ and the input gate $I_{t}$ on what new information to store in $c_{t}$.

The output gate $O_{t}$ in (10) determines how much of the updated cell state $c_{t}$ should be exposed as $h_{t}$, which represents the hidden state computed based on the output gate $O_{t}$ and the updated cell state $c_{t}$ as in (11), such that tanh() is the hyperbolic tangent activation function.

(10) $$O_{t}=\sigma \left(W_{o}\left[h_{t-1},x_{t}\right]+b_{o}\right)$$

(11) $$h_{t}=O_{t}\cdot \tanh\left(c_{t}\right)$$

#### 2.3.4. Attention Mechanism

The attention mechanism allows the model to focus on the most relevant parts, which are the selected features of the input sequence by computing attention scores. These scores determine the importance of each element in the sequence, enabling the model to highlight and prioritize important features. The context vector produced by the attention mechanism is a weighted summary of the input sequence [59]. The input to the attention mechanism is the output sequence from the LSTM layer $h_{t}$ for t=1,2,…,T, where *T* is the length of the sequence. The attention mechanism first computes a set of attention scores $e_{t}$ for each time step *t* based on (12):

(12) $$e_{t}=\tanh\left(W\cdot h_{t}+b\right),$$

where *W* is a weight matrix, and *b* is a bias vector. The score $e_{t}$ can be seen as an intermediate representation that helps to determine the importance of each $h_{t}$. The scores $e_{t}$ are then normalized using a softmax function to obtain the attention weights $\alpha_{t}$. These weights sum to 1 and represent the relative importance of each time step.

(13) $$\alpha_{t}=\frac{\exp(e_{t})}{\sum_{k=1}^{T}\exp\left(e_{k}\right)},$$

such that $\alpha_{t}$ is the attention weight for time step *t*. The attention weights $\alpha_{t}$ are used to compute a weighted sum of the LSTM outputs, resulting in a context vector *C*:

(14) $$C=\sum_{t=1}^{T}\alpha_{t}\cdot h_{t},$$

where *C* represents the weighted combination of the LSTM hidden states.

#### 2.3.5. Skip Connection

The skip connection combines the output of the attention mechanism with the summed output of the LSTM layer as given in (15). This helps retain important information from the original sequence while also incorporating the focused information from the attention mechanism. Skip connections can improve the gradient flow and help in training deeper networks.

(15) $$\mathrm{Combined}\;\mathrm{Output}=C+\sum_{t=1}^{T}h_{t}$$

#### 2.3.6. Prediction Process

This is the final dense layer responsible for producing the classification output. The number of points taken into consideration in this process corresponds to the number of classes in the classification task. The softmax activation function ensures that the output is a probability distribution over the classes, such that

(16) $$Y=\mathrm{softmax}(W_{d}\cdot \mathrm{Combined}\;\mathrm{Output}+b_{d}),$$

where $W_{d}$ is the weight matrix of the dense layer, $b_{d}$ is the bias of the dense layer, and *Y* is the final output, which is a probability distribution over the classes. Algorithm A1, provided in Appendix A, summarizes the procedural steps followed by our proposed MediVision model, including input processing, feature extraction using CNNs, LSTM, attention mechanism, skip connection, and predication layers, respectively, to effectively analyze medical images and accurately classify diseases based on learned features.

In this study, we adopted a trial-and-error-based approach to tune hyperparameters, which is a commonly used strategy in DL-based models when automated search methods are found to be of high computational cost [50,56,60]. Specifically, we experimented with various configurations for key parameters such as the number of CNN layers, LSTM units, dropout rates, and learning rates, using validation performance as the guiding metric. Given the heterogeneous nature of the ten medical image datasets investigated in this study, with each having different imaging modality and class distribution, we observed that a uniform configuration often led to suboptimal results on certain datasets. To address this, we performed dataset-specific hyperparameter tuning, and we have documented the codes and final settings for each dataset to enable the reproducibility of our results. While our manual tuning approach yielded competitive results, we acknowledge that a more systematic strategy, such as grid search or Bayesian optimization, could further refine the performance and efficiency. We consider this a promising direction for our future work and plan to explore it in subsequent research efforts. Notably, recent studies, such as the work in [61], have demonstrated the effectiveness of Bayesian optimization in complex model tuning tasks.

## 3. Results

In this section, the numerical results of the proposed MediVision model are observed and discussed from different perspectives. First, we consider the Accuracy, which measures how often the model is correct across all classes. It is the ratio of the number of correct predictions considering True Positives (TP), True Negatives (TN), and the Total number of Instances (TI).

(17) $$Accuracy=\frac{TP+TN}{TI}$$

Precision measures the proportion of TP instances among all instances predicted as positive, which comprises TP and False Positives (FP). It indicates how many of the predicted positives are truly positives.

(18) $$Precision=\frac{TP}{TP+FP}$$

Recall measures the proportion of TP instances among all actual positive instances, which comprises TP and False Negatives (FN). It shows how well the model identifies positive instances.

(19) $$Recall=\frac{TP}{TP+FN}$$

The F1-Score is the harmonic mean of Precision and Recall, providing a balance between them.

(20) $$F1\text{-}Score=2\times \frac{Precision\cdot Recall}{Precision+Recall}$$

TPs are the instances that the model correctly identifies as positive, meaning both the prediction and the actual status are positive. TNs represent the instances that the model accurately predicts as negative, where both the prediction and the actual status are negative. Conversely, FPs occur when the model incorrectly predicts a positive outcome for an instance that is negative, while FNs arise when the model mistakenly predicts a negative outcome for an instance that is actually positive.

### 3.1. Alzheimer’s Disease Dataset

**Figure 4:**
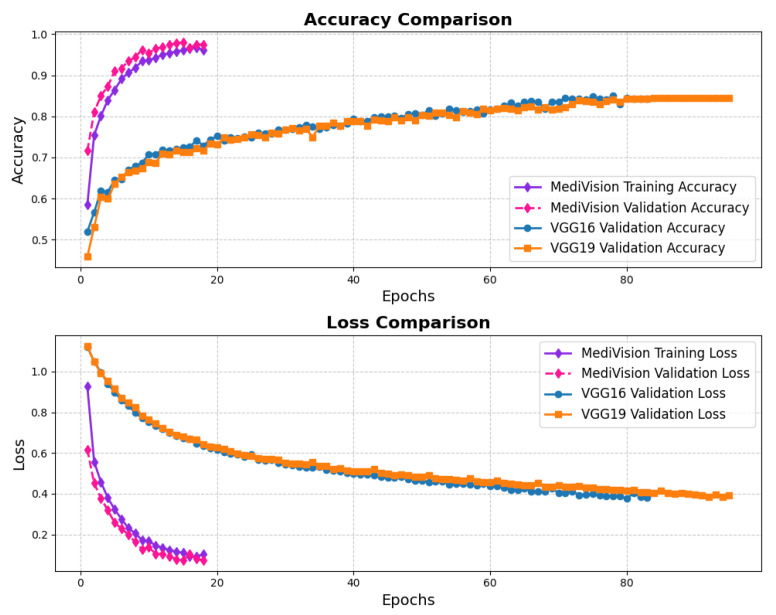
Accuracy and loss graph of Alzheimer’s disease dataset.

The assessment of the proposed MediVision model for Alzheimer’s disease classification was demonstrated through accuracy-loss trends, confusion matrix and key metrics such as Precision, Recall, and F1-Score. The accuracy and loss curves, illustrated in Figure 4, reveal a steady improvement in MediVision’s performance across training epochs. The training accuracy starts at approximately 60% and gradually increases, reaching around 95% by the final epoch. A similar trend is observed in validation accuracy, which consistently outperforms training accuracy at multiple points, ultimately stabilizing at approximately 96%. This close alignment between the training and validation accuracies suggests strong generalization capabilities, minimizing overfitting risks. Additionally, the loss curves indicate effective learning, with both training and validation losses consistently decreasing and stabilizing around 0.07, which is a large improvement compared to a 40% loss of both the VGG16 and VGG19 models. In addition, the smooth decline, without sharp fluctuations, demonstrates the efficient convergence of our proposed model.

**Figure 5:**
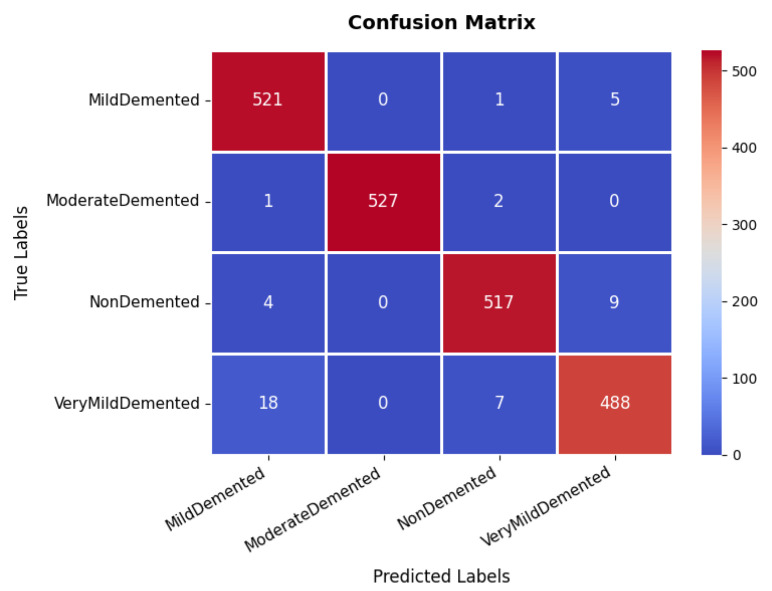
Confusion matrix of Alzheimer’s disease dataset.

The confusion matrix, which is illustrated in Figure 5, provides an in-depth analysis of the model’s classification performance across four categories: non-demented, very mild demented, mild demented, and moderate demented. The majority of samples are correctly classified, with 521, 527, 517, and 488 correctly classified instances, respectively. Although most samples are accurately classified, a minor misclassification trend is observed in the very mild demented category, where instances are occasionally misclassified as mild demented. This suggests possible feature similarities between these two conditions. However, the overall classification accuracy remains high, underscoring the model’s reliability in distinguishing between different dementia stages. The effectiveness of the model is further validated through the precision, recall, and F1-score metrics. The moderate demented category achieves an exceptional precision and recall of 100%, indicating perfect classification for this group. Similarly, other categories exhibit high performance, with precision values of 96% (mild demented), 98% (non-demented), and 97% (very mild demented). The recall scores remain consistently strong, with mild demented at 99%, moderate demented also at 99%, non-demented at 98%, and very mild demented at 96%. The F1-score, which represents a harmonic mean between precision and recall, follows a similar pattern, with an average score of 98%.

### 3.2. Blood Cell Dataset

**Figure 6:**
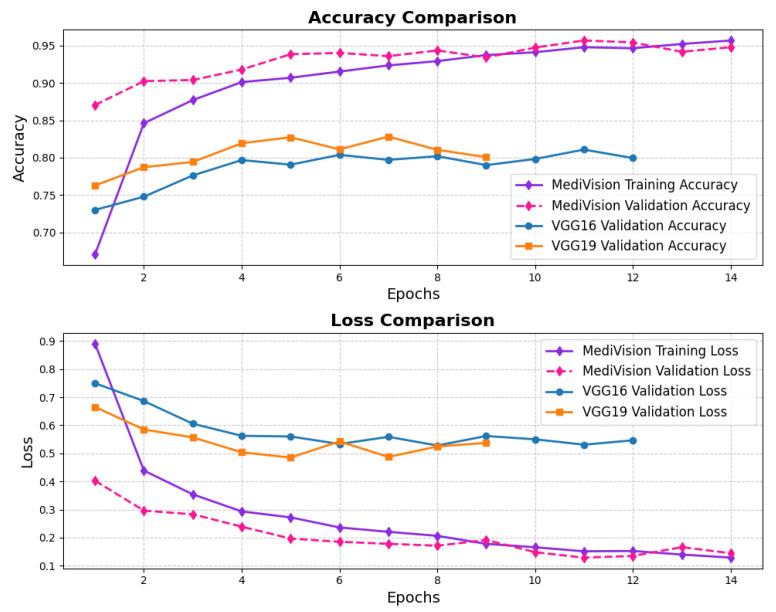
Accuracy and loss graph of blood cell dataset.

The performance evaluation for the blood cell image dataset follows a similar approach, with the accuracy and loss results depicted in Figure 6, the training accuracy exhibits a consistent upward trend, beginning at lower levels and converging to over 95%, while the validation accuracy follows a similar trajectory, ultimately stabilizing at around 95% with minimal signs of overfitting. The corresponding loss curves reinforce this stability, showing a smooth and steady decrease over the epochs.

**Figure 7:**
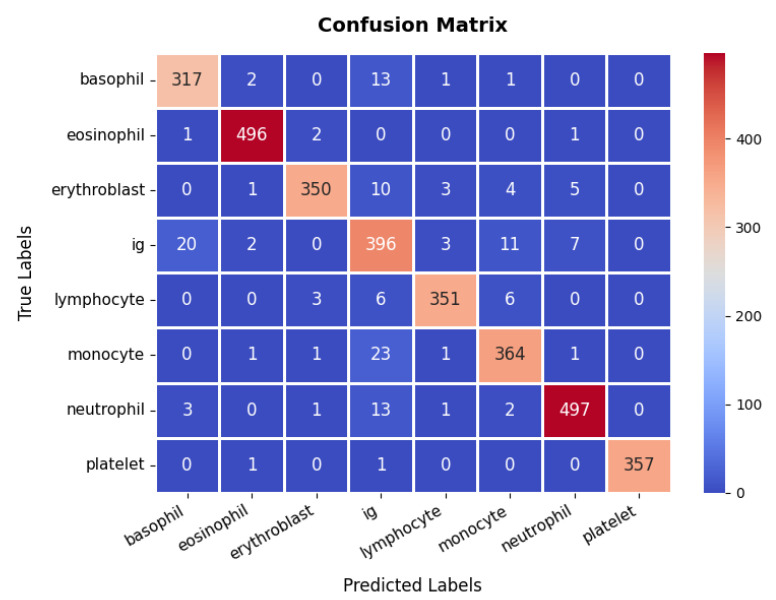
Confusion Matrix of Blood Cell dataset.

The confusion matrix, presented in Figure 7, reveals that the model accurately classifies the majority of samples across all cell types, including basophils, eosinophils, erythroblasts, Ig (immunoglobulins), lymphocytes, monocytes, neutrophils, and platelets, although occasional misclassifications occur, particularly among closely related classes such as Ig and monocytes. Still, the overall classification accuracy remains high, at approximately 95.45%. Furthermore, the detailed metrics underscore this effectiveness, with most classes attaining precision and recall values above 90%, resulting in F1-scores that consistently indicate strong performance. For instance, the eosinophil and neutrophil classes achieve near-perfect precision and recall, whereas Ig shows slightly lower metrics, suggesting a potential area for further refinement. Collectively, the high accuracy, well-balanced precision and recall, and smoothly converging loss function validate the model’s reliability in differentiating various blood cell types, thereby showcasing its utility in clinical diagnostic workflows.

### 3.3. Bone Fracture Multi-Region Dataset

**Figure 8:**
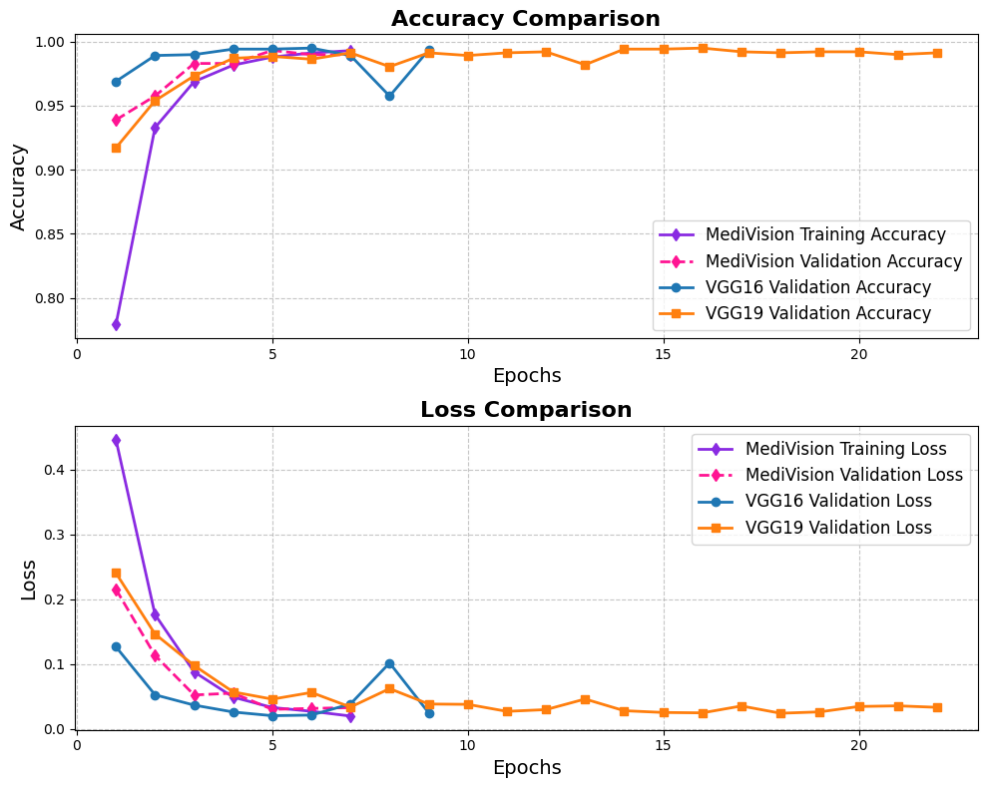
Accuracy and loss graph of bone fracture multi-region dataset.

The loss and accuracy curves for the bone fracture multi-region dataset, depicted in Figure 8, indicate high efficiency of our model in learning and generalization, where the training loss drops steeply from above 0.45 to nearly 0.05 within the first three epochs, demonstrating rapid learning, while the validation loss follows a similar declining trend, reaching below 0.05. By the end of training, both training and validation losses stabilize at very low values, signifying minimal errors. The accuracy curves further reinforce the model’s effectiveness, with the training accuracy rising sharply from approximately 80% to nearly 95% within the first few epochs, and the validation accuracy surpassing 95% by the second epoch, ultimately reaching nearly 98% at the final epoch with minimal overfitting and strong generalization.

**Figure 9:**
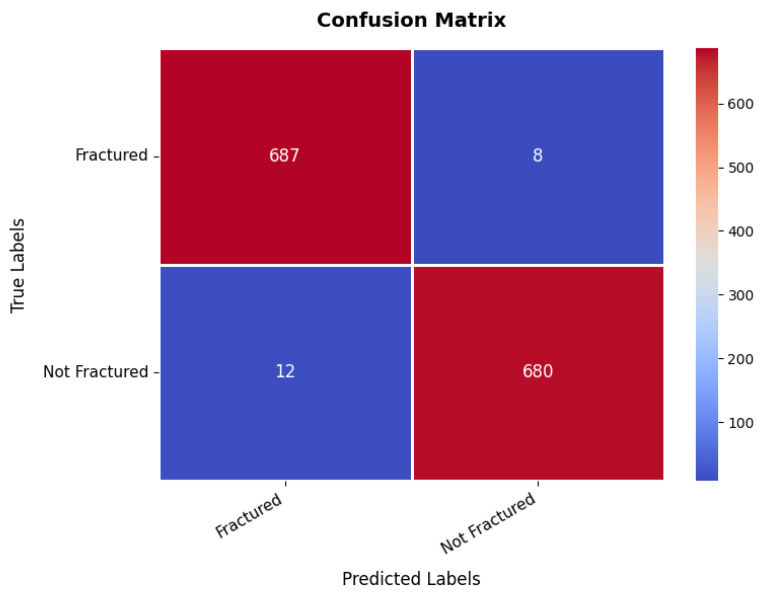
Confusion matrix of bone fracture multi-region dataset.

At the same time, the confusion matrix in Figure 9 provides additional validation, showing that the first class achieves 687 TPs with only eight misclassifications as the second class, while the second class records 680 TPs with just 12 false positives, demonstrating excellent classification performance. The small number of misclassifications highlights the model’s high predictive accuracy, making it suitable for real-world bone fracture detection. Furthermore, the precision, recall, and F1-score for each class range between 98% and 99%, with an overall model accuracy of 98.56%, confirming its exceptional reliability.

### 3.4. Brain Tumor Dataset

**Figure 10:**
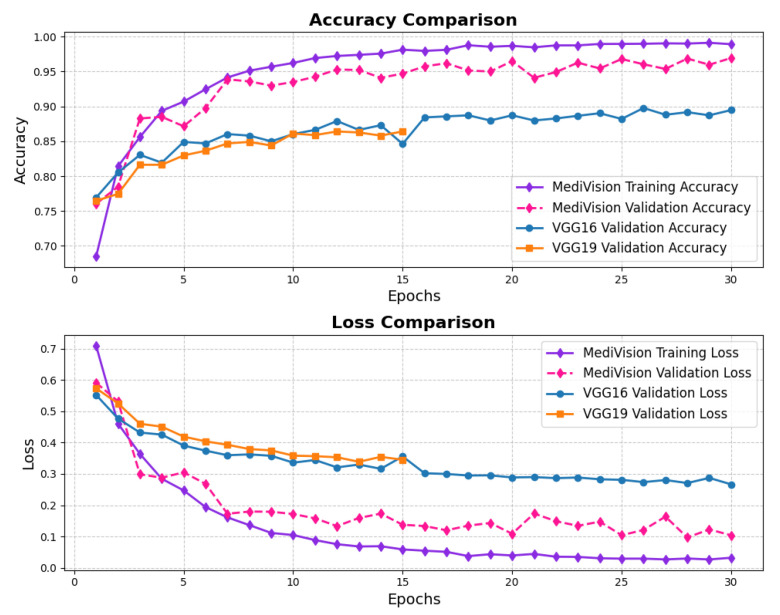
Accuracy and loss graph of brain tumor dataset.

The accuracy–loss curves for the brain tumor dataset are given in Figure 10, illustrating a consistent decrease in both training and validation loss over the epochs, with the training loss dropping sharply from approximately 0.7 to around 0.05 by the 20th epoch. This steep decline indicates that the model quickly learns the underlying patterns within the dataset. The validation loss follows a similar decreasing trend but remains slightly higher and fluctuates around 10% in later epochs, suggesting mild overfitting, where the model performs slightly better on training data than on unseen validation data. The accuracy curves further reinforce this observation, with the training accuracy rising steadily and reaching nearly 98%, while the validation accuracy, though slightly lower and exhibiting minor fluctuations, stabilizes around 95% by the final epochs.

**Figure 11:**
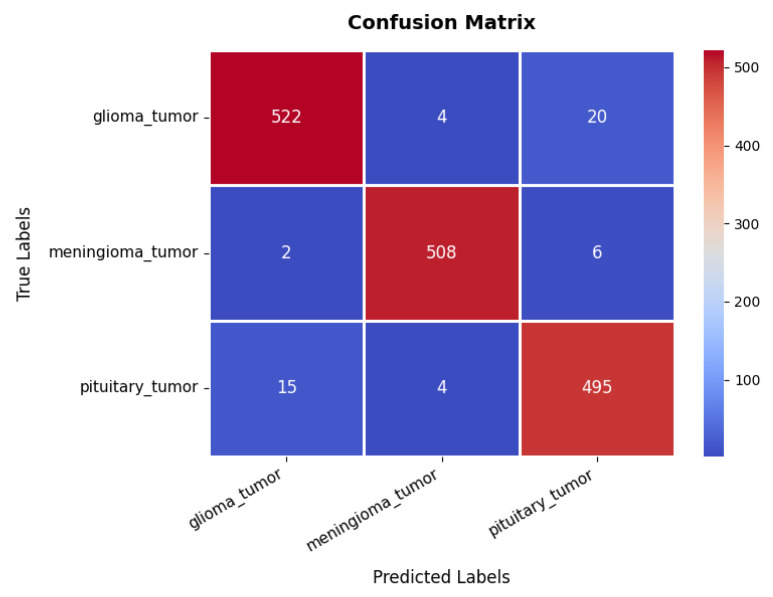
Confusion matrix of brain tumor dataset.

Looking at the confusion matrix in Figure 11, we get further insights into the model’s classification performance across different brain tumor classes. The glioma class achieves the highest classification accuracy, with 522 TPs and minimal misclassifications into other categories. Similarly, the meningioma and pituitary classes exhibit strong predictive accuracy, with 508 and 495 TPs, respectively, though some samples are misclassified due to overlapping features among the three tumor types. These classification results are further supported by the performance metrics in Table 3, where all three classes exhibit high precision, recall, and F1-scores ranging between 95% and 98%. The overall accuracy of the model stands at 96.76%, signifying its robustness in distinguishing between different tumor types.

### 3.5. Breast Ultrasound Dataset

**Figure 12:**
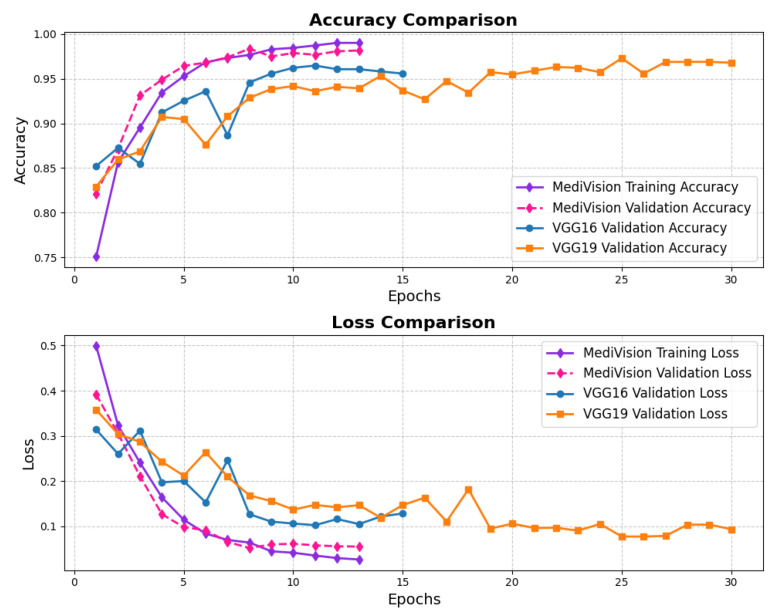
Accuracy and loss graph of breast ultrasound dataset.

The accuracy and loss trends for the case of breast ultrasound image classification are illustrated in Figure 12, in which we notice a steady improvement over the training epochs, indicating effective learning. Initially, the training accuracy starts at approximately 75%, gradually increasing to reach around 98% by the final epoch. The validation accuracy follows a similar trajectory, consistently outperforming the training accuracy at certain points and ultimately stabilizing at approximately 97%, with minimal the risk of overfitting. The loss curves further reinforce this stability, exhibiting a consistent decline without abrupt fluctuations, signifying smooth convergence.

**Figure 13:**
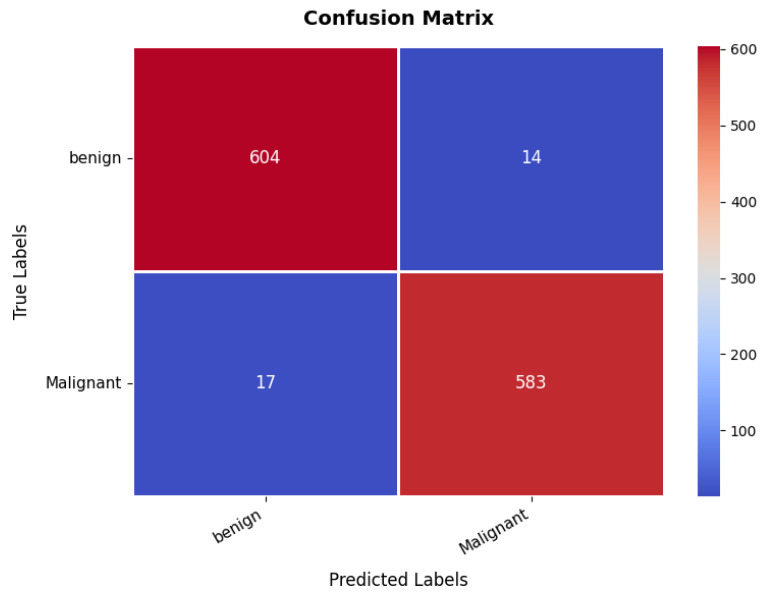
Confusion matrix of breast ultrasound dataset.

The confusion matrix, presented in Figure 13, provides insights into the classification performance for benign and malignant cases. The model correctly classifies 604 benign cases and 583 malignant cases. However, 14 benign samples are misclassified as malignant, while 17 malignant samples are misclassified as benign. Despite these minor misclassifications, the overall classification accuracy remains at an 97%, demonstrating the model’s robustness in distinguishing between benign and malignant breast tumors. Furthermore, the precision, recall, and F1-score metrics validate the model’s efficiency and reliability, as shown in Table 3. The benign class achieves a precision of 97%, recall of 98%, and  F1-score of 97%, indicating strong classification performance. Similarly, the malignant class exhibits a precision of 97.45% and recall of 98%, reflecting a balanced classification ability across both categories. The high recall values indicate that the model is highly effective in identifying malignant cases, which is critical for medical diagnosis.

### 3.6. Chest CT Scans Dataset

**Figure 14:**
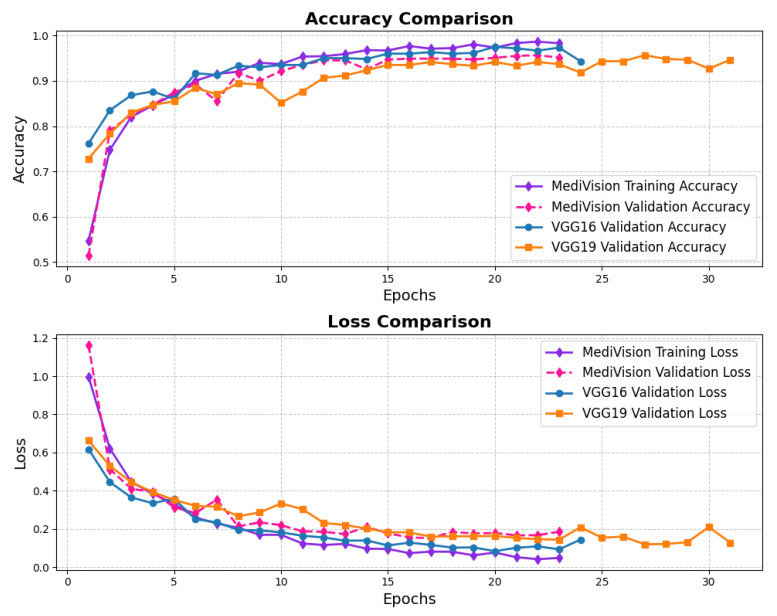
Accuracy and loss graph of chest CT scans dataset.

The loss and accuracy curves, as illustrated in Figure 14 demonstrate strong performance for the chest CT scans dataset. The training loss exhibits a substantial reduction from approximately 1 to 0.4 within the initial epochs, indicating a rapid learning phase. A similar trend is observed in the validation loss, which decreases from approximately 0.8 to 0.4, suggesting a well-balanced learning process and a reasonable level of generalization. As training progresses, both the training and validation losses continue to decline. Notably, the validation loss experiences fluctuations around epochs 10 and 14. However, in general, the losses remain balanced, affirming the model’s stability and robustness. The accuracy curves follow a similar pattern, reinforcing the effectiveness of the model. The training accuracy experiences a sharp rise from approximately 60% to over 90% within the first three epochs, indicating the model’s ability to quickly learn the dataset’s inherent patterns. The validation accuracy also improves steadily, closely following the training accuracy. After approximately five epochs, both the training and validation accuracy stabilize around 95%, with the training accuracy exhibiting minimal variation and the validation accuracy showing slight oscillations. These minor fluctuations in validation accuracy, reflected in the loss curves, indicate that the model maintains a well-calibrated performance without significant overfitting.

**Figure 15:**
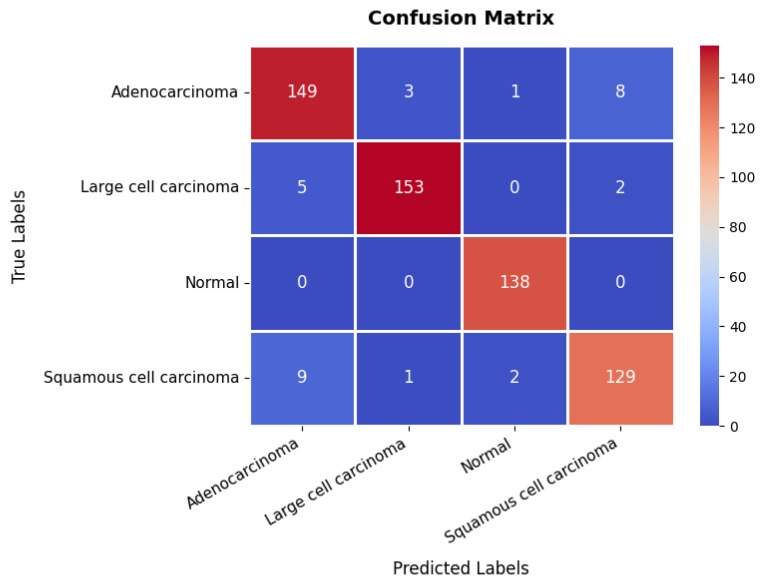
Confusion matrix of chest CT scans dataset.

The confusion matrix, presented in Figure 15, provides further insights into the model’s classification performance across the four classes. The first class achieves 149 TPs, though eight instances are misclassified as belonging to the fourth class. The second class is correctly classified in 153 instances, with only five misclassified as the first class, suggesting some overlap in features between these two categories. The third class is handled with high precision, achieving 138 TPs with no misclassifications, underscoring the model’s strong ability to distinguish this class. The fourth class records 129 TPs but exhibits minor misclassification errors, with nine instances predicted as the first class and two as the third class. The overall accuracy of the model on this dataset is 94.83%, as shown in Table 3. These results confirm the model’s reliability in distinguishing between different categories within the chest CT scan while maintaining a strong balance between training and validation performance.

### 3.7. Chest X-Ray Dataset

**Figure 16:**
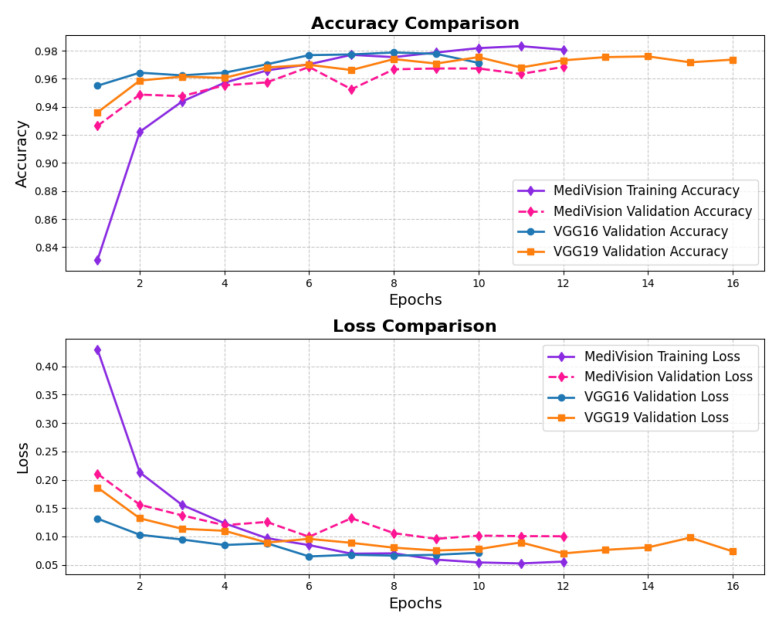
Accuracy and loss graph of chest X-ray dataset.

The performance evaluation for the chest X-ray dataset, also follows the same structured approach. The accuracy–loss curves illustrated in Figure 16 indicate that the training accuracy exhibits a consistent upward trajectory, starting at approximately 84% and steadily increasing to nearly 98% over the epochs. Similarly, the validation accuracy follows a comparable trend, stabilizing around 96–97% with minimal signs of overfitting. The corresponding loss curves further reinforce this stability, showing a steady decline in both training and validation loss. This confirms that the model trains efficiently and achieves robust convergence.

**Figure 17:**
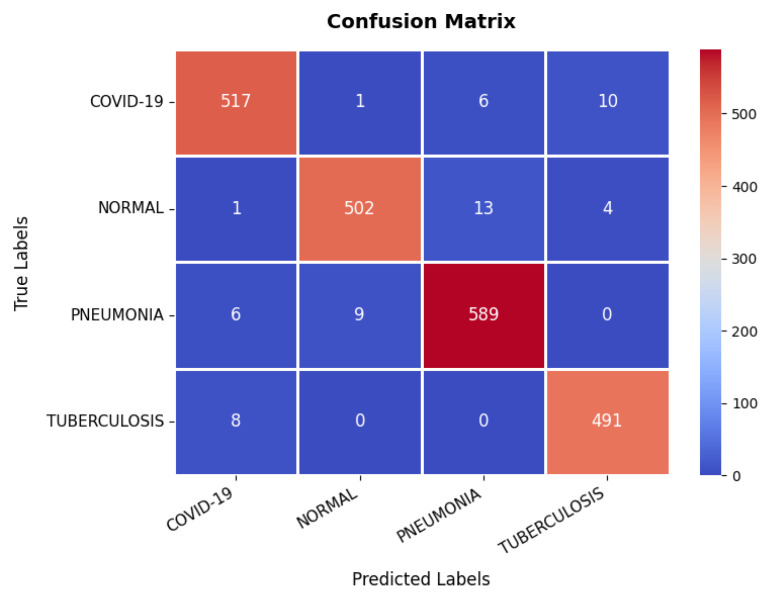
Confusion matrix of chest X-ray dataset.

Looking at the confusion matrix, which is presented in Figure 17, shows that the model classifies the majority of samples correctly across all categories: COVID-19, Normal, Pneumonia, and Tuberculosis. Notably, COVID-19 and Pneumonia classifications have a few misclassifications, with some Pneumonia cases being misclassified as Normal or COVID-19. However, the overall misclassification rate remains low, with a strong emphasis on correctly identifying disease conditions. The detailed evaluation metrics presented in Table 3 further validate the model’s high performance. The precision, recall, and F1-score values consistently exceed 97% across all classes, indicating a well-balanced classification model. The overall classification accuracy is 97.31%, confirming that the model is highly reliable in diagnosing chest-related conditions. While minor misclassifications exist, they do not significantly impact the overall performance, making the model highly suitable for clinical diagnostic applications.

### 3.8. Diabetic Retinopathy Dataset

**Figure 18:**
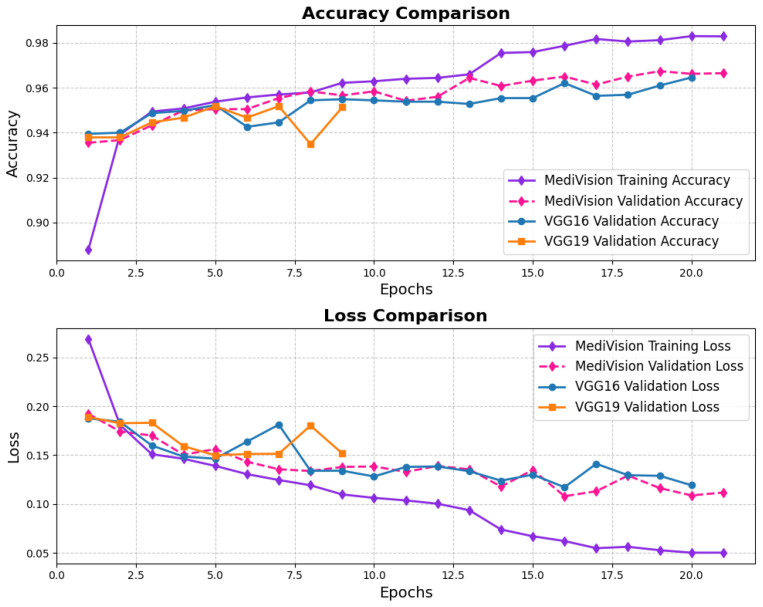
Accuracy and loss graph of diabetic retinopathy dataset.

The loss and accuracy curves for the diabetic retinopathy dataset, as illustrated in Figure 18, exhibit similar characteristics to those observed in other datasets. However, the proposed model demonstrates high efficiency despite some oscillations in validation performance. The training loss steadily decreases from approximately 0.28 to 0.12, indicating a consistent learning process, with all log loss values remaining below 0.05, suggesting that the model effectively learns and fits well to the training data. Although the validation loss follows a similar downward trend, it exhibits some fluctuations, particularly between epochs 3 and 6, before stabilizing and converging toward the training loss, demonstrating improved generalization. The training accuracy rapidly increases from approximately 88% to 94% within the first three epochs, signifying the model’s ability to quickly adapt to the dataset, while the validation accuracy also improves sharply, reaching around 95% by epoch 3, though it shows some minor oscillations between epochs 4 and 6, mirroring the validation loss. Nonetheless, by the final epochs, both the training and validation accuracy stabilize at approximately 95%, confirming that the model generalizes well without significant overfitting.

**Figure 19:**
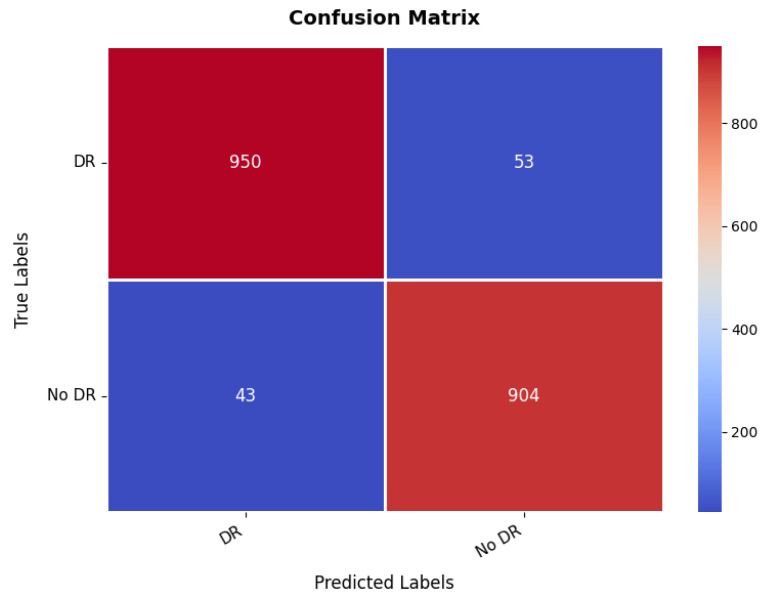
Confusion matrix of diabetic retinopathy dataset.

Additional insight in to the classification ability of the model is given by the confusion matrix, which is illustrated in Figure 19. The matrix indicates a TP value, which is much higher for both classes with 950 correct predictions for the first class, while 904 for the second class were correctly predicted. However, there are some misclassified, where 53 instances of the first class have been classified wrongly as the second class, and 43 instances of the second class were classified as the first class. These numbers are still low enough that it suggests the overall accuracy is good, but it also hints at some of the times where this model must get confused between the two classes, probably because the images making up the set of diabetic retinopathy are nuanced in their features. The balance between the TPs and the misclassifications suggests that while the model is highly effective, further refinement could help reduce these errors and improve the overall accuracy of the classification. The range of precision, recall, and F1-score is in between 95% and 96% for both the classes. The overall accuracy is 95.43%.

### 3.9. Kidney Diseases Dataset

**Figure 20:**
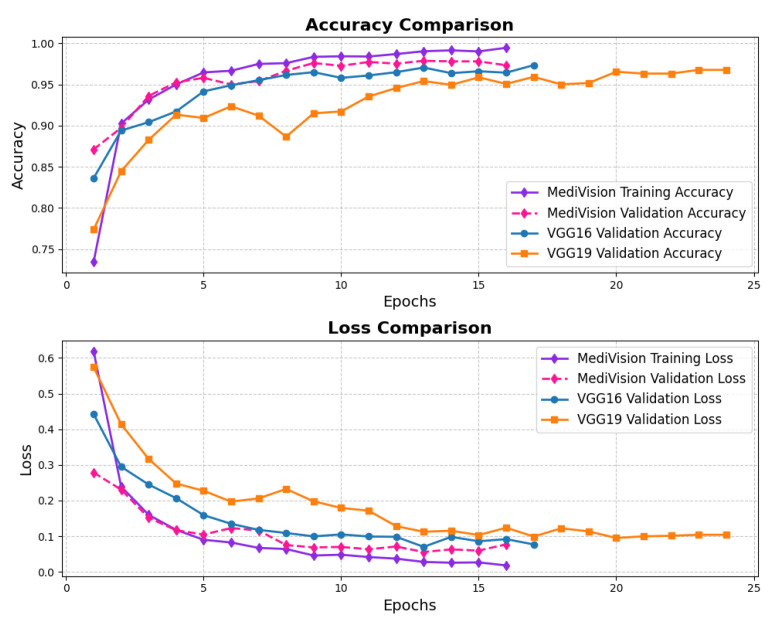
Accuracy and loss graph of kidney diseases dataset.

The loss and accuracy curves for the kidney diseases dataset, given in Figure 20, demonstrate a highly optimized model with strong generalization capabilities. The training loss decreases steadily from 0.6189 to 0.0180, while the validation loss shows some fluctuations, starting at 0.2786 and ending at 0.0771. The accuracy trends confirm the model’s strong performance. The training accuracy increases sharply from 75% to 95% within the first five epochs, showing the model’s quick adaptation, while the validation accuracy closely follows, occasionally surpassing the training accuracy, highlighting strong generalization. After the initial phase, the training accuracy stabilizes around 97% with minor fluctuations, while the validation accuracy oscillates slightly around 94%, maintaining high performance on both sets.

**Figure 21:**
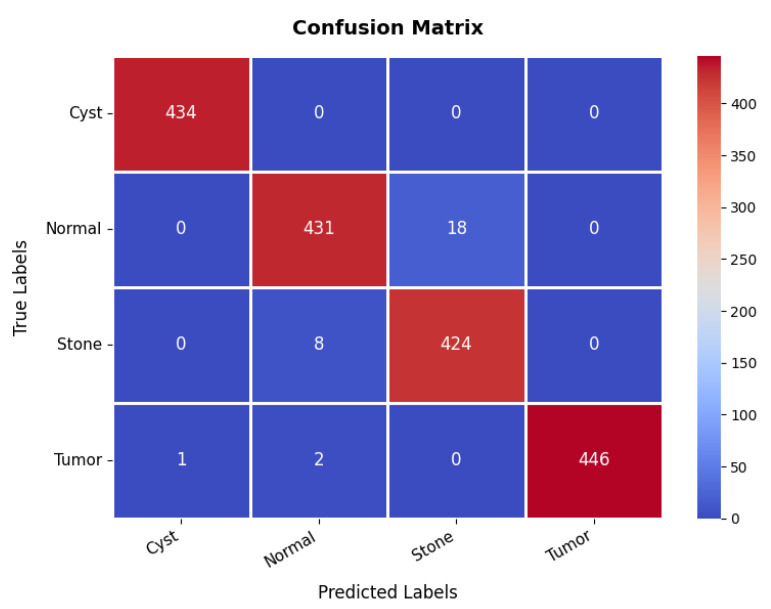
Confusion matrix of kidney diseases dataset.

The confusion matrix in Figure 21 further validates the model’s classification effectiveness across different classes. The first class achieves perfect classification with 434 TPs and no FPs. The second class is also well-classified, with 431 TPs, though 18 samples are misclassified as the third class, indicating some difficulty in distinguishing between these two classes. The third class has 424 TPs but shows eight misclassifications as the second class, reinforcing this challenge. The fourth class exhibits excellent classification, with 446 TPs and only three minor misclassifications, with two instances misclassified as the second class. Overall, the model demonstrates strong classification performance, with occasional confusion between the second and third classes. The overall accuracy for the kidney diseases dataset is 98.36%, as summarized in Table 3.

### 3.10. Retinal OCT Dataset

**Figure 22:**
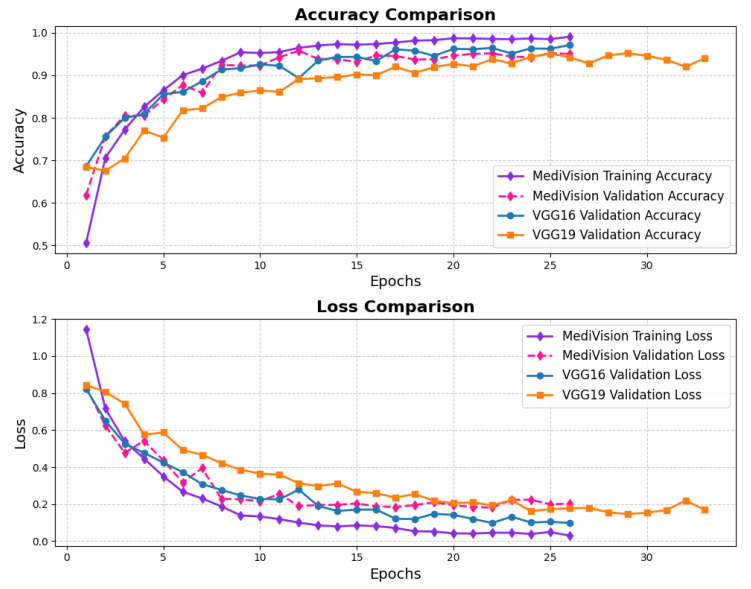
Accuracy and loss graph of retinal OCT dataset.

The loss curves for the retinal OCT dataset are presented in Figure 22, showing a significant and consistent reduction in training loss, which declines from approximately 1.2 to nearly 0.01 by the 25th epoch. The validation loss follows a similar decreasing trend; however, it exhibits minor fluctuations, particularly between the fifth and tenth epochs. This variability in validation loss suggests that, while the model effectively learns feature representations, some inconsistencies in the validation set may contribute to occasional performance fluctuations. Despite these variations, the overall decline in validation loss indicates enhanced generalization capability, and both the training and validation losses eventually converge at low values, signifying a stable and well-optimized model. The accuracy curves further support the model’s strong performance, demonstrating a rapid increase in training accuracy, surpassing 90% within the first five epochs and continuing to improve steadily. The validation accuracy follows a comparable trajectory, albeit slightly lower than the training accuracy, with minor fluctuations mirroring those observed in validation loss. By the 25th epoch, both the training and validation accuracies stabilize at approximately 95%, which indicates minimal overfitting.

**Figure 23:**
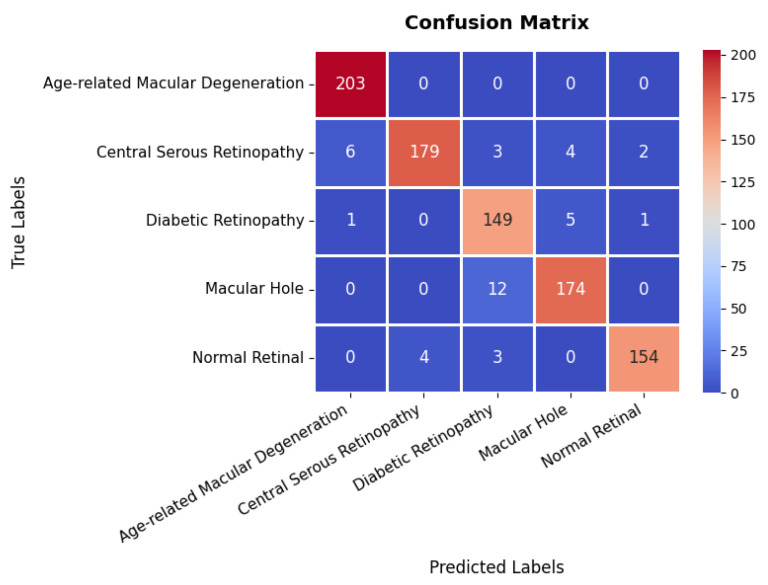
Confusion matrix of retinal OCT dataset.

The confusion matrix in Figure 23 provides a detailed evaluation across different classes in the retinal OCT dataset. The first class exhibits near-perfect classification, achieving 203 TPs with no observed misclassifications, highlighting the model’s high precision for this category. The second class also demonstrates strong classification performance with 179 TPs; however, six instances are misclassified as the first class, and a few others are incorrectly assigned to different categories. The third class, with 149 TPs, shows a slightly higher misclassification rate, with certain instances incorrectly categorized as either the second or fourth class. The fourth class attains 174 TPs with minimal classification errors, primarily involving minor confusion with the third class. Overall, the model exhibits robust classification performance, with only minor misclassifications observed in the second and third classes, effectively distinguishing between the majority of classes and providing high precision and recall as summarized in Table 3.

## 4. Discussion

### 4.1. Model Interpretability via Explainable AI

**Figure 24:**
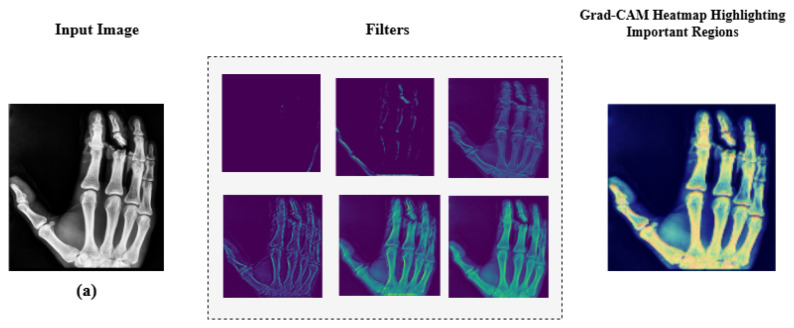

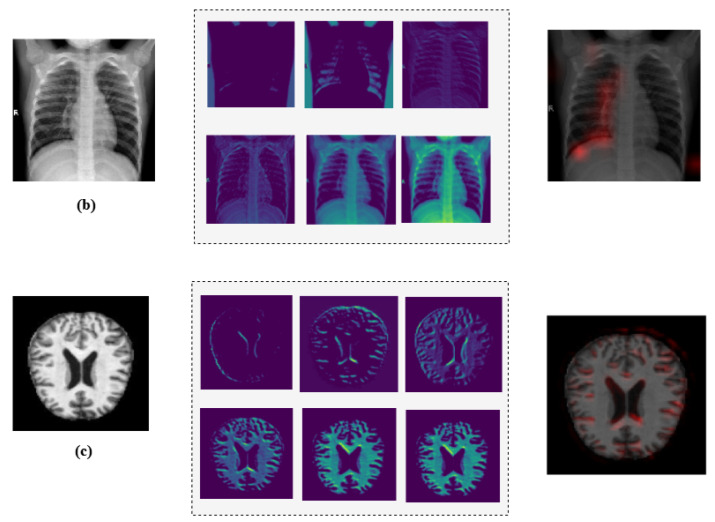
Grad-CAM heatmap visualization showing important regions (in red) for disease classification across representative datasets: (**a**) bone fracture multi-region, (**b**) chest X-ray, and (**c**) Alzheimer’s disease.

Additionally, we employed Grad-CAM heatmaps to visually explain MediVision’s decision-making process across representative medical imaging datasets, namely (a) bone fracture multi-region, (b) chest X-ray, and (c) Alzheimer’s disease, as illustrated in Figure 24. These heatmaps highlight the image regions that contribute most significantly to the model’s class predictions. Since MediVision performs classification rather than segmentation, the Grad-CAM heatmaps emphasize discriminative features learned by the model, rather than anatomically precise regions of interest. The intermediate convolutional layers act as feature detectors, and the resulting activations, visualized as intensity-based overlays, reveal where the model focuses during classification. This visual interpretation validates that MediVision accurately identifies significant disease characteristics within the input image. The consistency of highlighted regions across these representative datasets supports the model’s generalizability and its ability to detect subtle disease patterns across diverse imaging modalities. These findings demonstrate MediVision’s potential to enhance diagnostic accuracy and reduce human error in clinical settings.

### 4.2. Comparative Analysis

The performance comparison between the proposed MediVision model and existing DL-based models, including VGG16, VGG19, and ResNet50, across all ten diverse medical image datasets is summarized in Table 4. Notably, MediVision showed substantial improvements in Alzheimer’s disease, achieving 98% compared to the 75–85% range of the other models. The results also demonstrated significant gains in blood cell classification with 95.45% accuracy and brain tumor detection reaching 96.76%, a considerable advancement over ResNet50’s by 24.44%. Significant gains were also observed in breast ultrasound, chest X-ray/CT, and kidney disease classification. For retinal OCT classification, while the benchmark models already achieve high accuracy, MediVision still demonstrates a considerable improvement in comparison.

In addition, Table 5 demonstrates MediVision’s competitive edge against state-of-the-art DL-based models, including EfficientNet, DenseNet, and Hybrid CNN-LSTM, across diverse medical image datasets. The table shows that MediVision is capable of achieving superior or comparable accuracy in nearly all categories, as a result of its advanced architecture and learning capabilities, particularly for classifying complex medical images like brain tumor and Alzheimer’s disease. These results were achieved through a consistent training strategy based on empirical tuning, where a common set of hyperparameters was identified using a hit-and-trial method. Specifically, we used a batch size of 16, a learning rate of 0.01 with the Adam optimizer, and dropout rates between 0.1 and 0.3. Additionally, early stopping with a patience of three epochs was applied, which helped ensure strong performance across multiple datasets. Overall, the results confirm MediVision’s enhanced generalization and accuracy, highlighting its potential for robust and reliable medical image analysis and disease diagnosis.

### 4.3. Statistical Analysis

To validate the effectiveness of the proposed MediVision hybrid CNN–LSTM model, statistical significance testing was performed against three baseline convolutional neural network architectures such as VGG16, VGG19, and ResNet50 across ten diverse medical imaging datasets. As summarized in Table 6, MediVision achieved the highest mean classification accuracy of 96.89%, outperforming VGG16 (92.98%), VGG19 (92.79%), and ResNet50 (81.17%). These consistent improvements demonstrate the superior generalization and stability of MediVision across multiple medical imaging modalities.

Pairwise comparisons between MediVision and each baseline model were conducted using both the paired *t*-test and the non-parametric Wilcoxon signed-rank test, as summarized in Table 7. The Wilcoxon tests yielded statistically significant results (*p* < 0.01) for all comparisons, confirming that the observed improvements were not due to random variation. Although the paired *t*-test for the comparison with VGG16 was marginal (*p* = 0.0597), the non-parametric Wilcoxon result confirmed significance. Furthermore, after applying the Holm–Bonferroni correction to control for multiple comparisons, all adjusted *p*-values remained below 0.01, reinforcing the robustness of the findings.

Finally, an overall comparison among all four models was performed using the Friedman test, a non-parametric alternative to repeated-measures ANOVA. As shown in Table 8, the test produced a $\chi^{2}$ value of 27.00 with a *p*-value of 0.000006 (*p* < 0.001), indicating a highly significant difference in performance across models. These results collectively demonstrate that MediVision provides statistically and practically significant improvements over conventional CNN architectures for medical image classification tasks.

## 5. Conclusions

This study introduces a hybrid framework for medical image classification that seamlessly integrates CNNs, LSTMs, and an attention mechanism with a skip connection to enhance the feature extraction and classification accuracy. Trained and tested on ten diverse representative medical image datasets, MediVision outperforms widely used pretrained models such as VGG16, VGG19, and ResNet50 by 3.91%, 4.10%, and 15.72%, respectively, in terms of the mean classification accuracy. The results of both paired statistical tests and the Friedman test confirm that these improvements are statistically significant (*p* = 0.000006) across multiple medical imaging modalities. The model’s strength lies in its ability to effectively capture both spatial and temporal features, with CNNs extracting spatial information, LSTMs learning sequential dependencies from image sequences, and the attention mechanism refining these features by focusing on the most relevant regions. The inclusion of a skip connection ensures efficient feature propagation, further boosting classification performance. To enhance the model transparency and support the clinical interpretability, Grad-CAM heatmaps were employed to visually highlight the critical image regions influencing classification decisions. Additionally, the datasets were carefully augmented and balanced, ensuring optimal generalization and robustness across different medical imaging domains.

For future work, we plan to conduct a detailed analysis of the computational complexity and runtime performance to evaluate MediVision’s scalability in real-world clinical environments. A comprehensive ablation study will also be undertaken to assess the individual and combined contributions of key architectural components (i.e., CNN, LSTM, attention mechanism, and skip connection). Furthermore, we aim to enhance the feature extraction and interpretability by exploring self-supervised learning and transformer-based architectures. To improve the generalizability, we will extend MediVision to multi-modal medical data, such as integrating MRI, CT scans, and histopathological images. In addition, future work will explore the application of federated learning to facilitate collaborative model training across decentralized medical institutions while preserving data privacy and the integration of MediVision into clinical decision support systems to enable real-time AI-driven diagnostics.

## Figures and Tables

**Table 1:** Summary of the datasets used for training and evaluation.

Dataset Name	Modality	No. of Images	Classes	Subcategories
Alzheimer’s Disease [38]	MRI	14,000	4	Non-demented, Very Mild,
				Mild, Moderate
Breast Ultrasound [39]	Ultrasound	8148	2	Benign, Malignant
Blood Cells [40]	Microscopic	21,841	8	Erythroblast, Neutrophil, Basophil, Eosinophil, Lymphocyte,
				Monocyte, Immature Granulocyte, Lymphocyte
Chest X-ray [41,42,43,44,45]	X-ray	14,375	4	Normal, COVID-19, Pneumonia, Tuberculosis
Chest CT Scans [46]	CT	4000	4	Normal, Adenocarcinoma, Large Cell, Squamous Cell
Diabetic Retinopathy [47]	Fundus	13,000	2	Diabetic, Non-Diabetic
Kidney Diseases [48,49]	Ultrasound	11,756	4	Normal, Cyst, Stone, Tumor
Bone Fracture [50]	X-ray	9206	2	Fractured, Non-Fractured
Retinal OCT [51]	OCT	6000	5	Normal, AMRD, CSR, DR, MH
Brain Tumor [52]	MRI	10,500	3	Glioma, Meningioma, Pituitary

**Table 2:** Data augmentation parameters.

Augmentation Technique	Description
Rotation	Rotates the image by fixed angles (90°, 180°, 270°).
Horizontal flip	Flips the image along the vertical axis.
Vertical flip	Flips the image along the horizontal axis.
Brightness adjustment	Randomly increases or decreases brightness. Factor range: 0.7–1.3.
Contrast adjustment	Randomly enhances or reduces contrast. Factor range: 0.8–1.5.

**Table 3:** Performance of MediVision model across subclasses of all medical image datasets.

Datasets	Subclasses	Precision (%)	Recall (%)	F1-Score (%)	Accuracy (%)
Alzheimer’s disease	Mild Demented	96.00	99.00	97.00	98.00
	Moderate Demented	100.00	99.00	100.00	
	Non-Demented	98.00	98.00	98.00	
	Very Mild Demented	97.00	95.00	96.00	
Breast ultrasound	Benign	97.00	98.00	97.00	97.45
	Malignant	98.00	97.00	97.00	
Blood cell	Basophil	93.00	95.00	94.00	95.45
	Eosinophil	99.00	99.00	99.00	
	Erythroblast	98.00	94.00	96.00	
	Ig	86.00	90.00	88.00	
	Lymphocyte	97.00	96.00	97.00	
	Monocyte	94.00	93.00	93.00	
	Neutrophil	97.00	96.00	97.00	
	Platelet	100.00	100.00	100.00	
Chest X-ray	COVID19	97.00	97.00	97.00	97.31
	Normal	98.00	97.00	97.00	
	Pneumonia	97.00	98.00	97.00	
	Tuberculosis	97.00	98.00	98.00	
Chest CT scans	Adenocarcinoma	91.00	93.00	92.00	94.83
	Large cell carcinoma	97.00	96.00	97.00	
	Normal	98.00	100.00	99.00	
	Squamous cell carcinoma	93.00	91.00	92.00	
Diabetic retinopathy	DR	96.00	95.00	95.00	95.43
	No DR	94.00	95.00	95.00	
Kidney diseases	Cyst	100.00	100.00	100.00	98.36
	Normal	98.00	96.00	97.00	
	Stone	96.00	98.00	97.00	
	Tumor	100.00	99.00	100.00	
Bone fracture	Fractured	98.00	99.00	99.00	98.56
	Not Fractured	99.00	98.00	99.00	
Retinal OCT	Age-related Macular Degeneration	97.00	100.00	98.00	95.44
	Central Serous Retinopathy	98.00	92.00	95.00	
	Diabetic Retinopathy	89.00	96.00	92.00	
	Macular Hole	95.00	94.00	94.00	
	Normal	98.00	96.00	97.00	
Brain tumor	Glioma	97.00	98.00	96.00	96.76
	Meningioma	98.00	96.00	98.00	
	Pituitary	95.00	98.00	96.00	

**Table 4:** Accuracy benchmarking of the proposed MediVision model.

Dataset	Model	Accuracy (%)
Alzheimer’s disease	VGG16	85.13
	VGG19	84.03
	ResNet50	75.13
	MediVision	98.00
Blood cell	VGG16	80.20
	VGG19	82.73
	ResNet50	69.09
	MediVision	95.45
Breast ultrasound	VGG16	96.47
	VGG19	97.29
	ResNet50	84.65
	MediVision	97.45
Chest X-ray	VGG16	96.00
	VGG19	96.30
	ResNet50	92.12
	MediVision	97.31
Chest CT scans	VGG16	96.00
	VGG19	95.67
	ResNet50	80.83
	MediVision	96.17
Diabetic retinopathy	VGG16	95.21
	VGG19	95.18
	ResNet50	89.13
	MediVision	95.43
Kidney diseases	VGG16	97.05
	VGG19	96.54
	ResNet50	90.08
	MediVision	98.36
Bone fracture multi-region	VGG16	98.42
	VGG19	98.13
	ResNet50	93.80
	MediVision	98.56
Retinal OCT	VGG16	95.44
	VGG19	95.22
	ResNet50	64.56
	MediVision	95.44
Brain tumor	VGG16	89.90
	VGG19	86.79
	ResNet50	72.32
	MediVision	96.76

**Table 5:** Accuracy comparison of MediVision against existing methods across medical image datasets.

Dataset	Reference	Method	Benchmark (%)	Proposed (%)
Alzheimer’s disease	Sethuraman et al. [60]	CNN	96.61	98.00
Breast ultrasound	Raptis et al. [39]	ResNet-50	93.05	97.45
Blood cell	Asghar et al. [62]	EfficientNet	94.70	95.45
Chest X-ray	Singh et al. [63]	VGG-16	94.70	97.31
Chest CT scans	Mamun et al. [64]	DenseNet	92.00	94.83
Diabetic retinopathy	Wang et al. [65]	InceptionV3	91.10	95.43
Kidney diseases	Bhandari et al. [48]	MobileNet	99.39	98.36
Bone fracture multi-region	Aldhwani et al. [50]	Xception	97.35	98.56
Retinal OCT	Dai et al. [66]	AlexNet	95.00	95.44
Brain tumor	Khan et al. [67]	Hybrid CNN-LSTM	95.10	96.76

**Table 6:** Mean accuracy across different models.

Model	Mean Accuracy (%)
VGG16	92.98
VGG19	92.79
ResNet50	81.17
MediVision	96.89

**Table 7:** Statistical significance analysis of MediVision compared with baseline models.

Comparison	Mean Difference (%)	Paired *t*-Test (*p*)	Wilcoxon (*p*)	Holm Adj. (*p*)
MediVision vs. VGG16	+3.91	0.0597	0.0039	0.0039
MediVision vs. VGG19	+4.10	0.0489	0.0020	0.0059
MediVision vs. ResNet50	+15.72	0.0006	0.0020	0.0039

**Table 8:** Overall statistical comparison of all models using the Friedman test.

Test	$\chi^{2}$	*p*-Value
Friedman test	27.00	0.000006

## Data Availability

The codes and datasets used within this study are made available through the public repository Zenodo at https://doi.org/10.5281/zenodo.15498860.

## Appendix A

The following algorithm summarizes the workflow of the proposed MediVision model.

**Algorithm A1** Proposed MediVision Procedure
**Input:** Image *I* with three channels (H,W,C), number of CNN layers *N*, LSTM units *U*, and number of classes *K*; 1: **Step 1—CNN Feature Extraction:** 2: **for** i=1 to *N* **do** 3: x← Conv2D$\left(filters_{i},\left(3,3\right),\mathrm{ReLU}\right)$ on *I*; 4: x← MaxPooling2D(2,2); 5: x← Dropout$\left(dropout_{i}\right)$; 6: **end for** 7: x← Flatten(x); 8: **Step 2—Reshape for LSTM:** 9: x← Reshape(1,−1); 10: **Step 3—LSTM Processing:** 11: $lstm_{out}\leftarrow$ LSTM(U,return_sequences = True) on *x*; 12: $lstm_{out}\leftarrow$ Dropout$\left(dropout_{lstm}\right)$; 13: **Step 4—Attention Mechanism:** 14: $attention_{out}\leftarrow$ Attention$\left(lstm_{out}\right)$; 15: **Step 5—Skip Connection:** 16: ${combined}_{out}$← Add(${attention}_{out}$, ∑ ${lstm}_{out}$); 17: **Step 6—Final Classification Layer:** 18: y← Dense(K,softmaxorsigmoid) on $combined_{out}$; 19: **Step 7—Compilation and Training:** 20: Loss: Sparse Categorical Crossentropy (multiclass) or Binary Crossentropy (binary); 21: Optimizer: Adam; 22: Metrics: Accuracy; 23: Apply Model Checkpoint and Early Stopping Callbacks;
**Output:** Classified label *y*;
